# Circular RNA circ_0000423 regulates cartilage ECM synthesis via circ_0000423/miRNA-27b-3p/MMP-13 axis in osteoarthritis

**DOI:** 10.18632/aging.204018

**Published:** 2022-04-19

**Authors:** Xing Li, Chaofan Xie, Fangjun Xiao, Haitao Su, Zhen Li, Jiaxian Weng, Yongming Huang, Peiheng He

**Affiliations:** 1 Department of Orthopedic Surgery, The Second Affiliated Hospital of Guangzhou University of Chinese Medicine, Guangzhou, Guangdong, China; 2 Guangzhou University of Chinese Medicine, Guangzhou, Guangdong, China; 3 Lingnan Medical Research Center of Guangzhou University of Chinese Medicine, Guangzhou, Guangdong, China; 4 Department of Orthopedic Surgery, The Eighth Affiliated Hospital of Sun Yat-Sen University, Shenzhen, Guangdong, China; 5 Department of Joint Surgery, The First Affiliated Hospital of Sun Yat-Sen University, Guangzhou, Guangdong, China

**Keywords:** osteoarthritis, circ_0000423, miRNA-27b-3p, MMP-13, intra-articular injection

## Abstract

Circular RNA (circRNA) is related to many human diseases including osteoarthritis (OA). Our research purpose was to show that functional circRNAs have a role in the pathogenesis of OA, while also identifying potential circRNA that bind to miRNA-27b-3p. Microarray analysis was used to evaluate the expression of CircRNA in OA and normal cartilage. The role and functional mechanism of Circ_0000423 up-regulation were detected in OA and verified *in vitro* and *in vivo*. RNA transfection, qRT-PCR, Western blot analysis, immunofluorescence, and dual-luciferase assays were used to investigate the interaction between Circ_0000423 and miRNA-27b-3p *in vitro*. The roles of Circ_0000423 were discussed *in vivo*. Our results discovered 11 down-regulated circRNAs and 101 up-regulated circRNAs between control and OA tissues, and confirmed that Circ_0000423 an increase significantly in OA tissues by evaluating the different circRNAs expressions. Meanwhile, luciferase analysis confirmed Circ_0000423 can be directly targeted by miRNA-27b-3p and act as a miRNA-27b-3p sponge. Circ_0000423 can influence MMP-13 and collagen II expression by targeting miRNA-27b-3p expression as ceRNA in OA. Furthermore, AAV-shRNA-Circ 0000423 intra-articular injection slows the progression of OA by decreasing articular cartilage destruction and erosion, joint surface fibrosis, osteophyte formation, MMP-13 expression, and increasing collagen II expression in the articular cartilage of ACLT-induced OA mice model. These findings confirmed that the Circ_0000423-miRNA-27b-3p-MMP-13 axis could affect the pathogenesis of OA which might lead to a novel target for diagnostic molecular biological indicators and potential OA treatments.

## INTRODUCTION

Osteoarthritis (OA) is a post-traumatic or age-related chronic degenerative joint disease that is characterized by degradation of the articular cartilage, synovial membrane inflammation, and subchondral bone sclerosis as well as the formation of osteophyte [1, 2]. There are different risk factors including previous joint trauma, aging, sex, or metabolic in OA [3, 4]; however, the molecular mechanisms of OA are still not fully understood, resulting in a lack of therapeutic approaches to OA. Hence, it’s vital to research and elucidate the pathogenesis mechanisms in OA, which may lead to the development of new and effective therapies for the treatment of OA.

Circular RNA (circRNA), the newly endogenous non-coding RNA (ncRNA), is featured for covalently closed-loop structures with neither polyadenylated tail nor 5′ to 3′ polarity [5, 6]. These structures lead to circRNA being more stable than linear RNAs *in vivo* [7]. Recently, more and more studies showed that circular RNA plays vital functions to regulate the development and progression of OA, and one of these vital functions is that they exert the “sponge” function to combine with miRNA to control the progression of OA [8–10]. MiRNAs, small non-coding RNAs with 20–22 nt in length, play an important role in regulating mRNA targets [11, 12]. Previous studies have shown that miRNA-27b-3p interacts with the 3′UTR of MMP-13(matrix metalloproteinase-13) mRNA and plays critical parts in the OA pathogenesis and progression [13]. The potential circRNAs that function as a “sponge” to combine with miRNA-27b-3p and regulate OA development, on the other hand, are unknown.

As a result, the present work aimed to discover putative circRNAs that bind to miRNA-27b-3p while also showing the significance of functional circRNAs in the pathogenesis of OA. In our studies, microarray and bioinformatics analysis were used to explore functional circRNAs on the pathogenesis of OA, as well as circRNA_0000423 was identified promoted MMP-13 expression through binding to miRNA-27b-3p. Our findings might lead to a novel technique for better understanding the pathogenesis of OA, and circRNA_0000423 could be employed as an effective and potentially therapeutic OA target.

## RESULTS

### Expression of circRNAs in control and OA specimens

**Figure 1:**
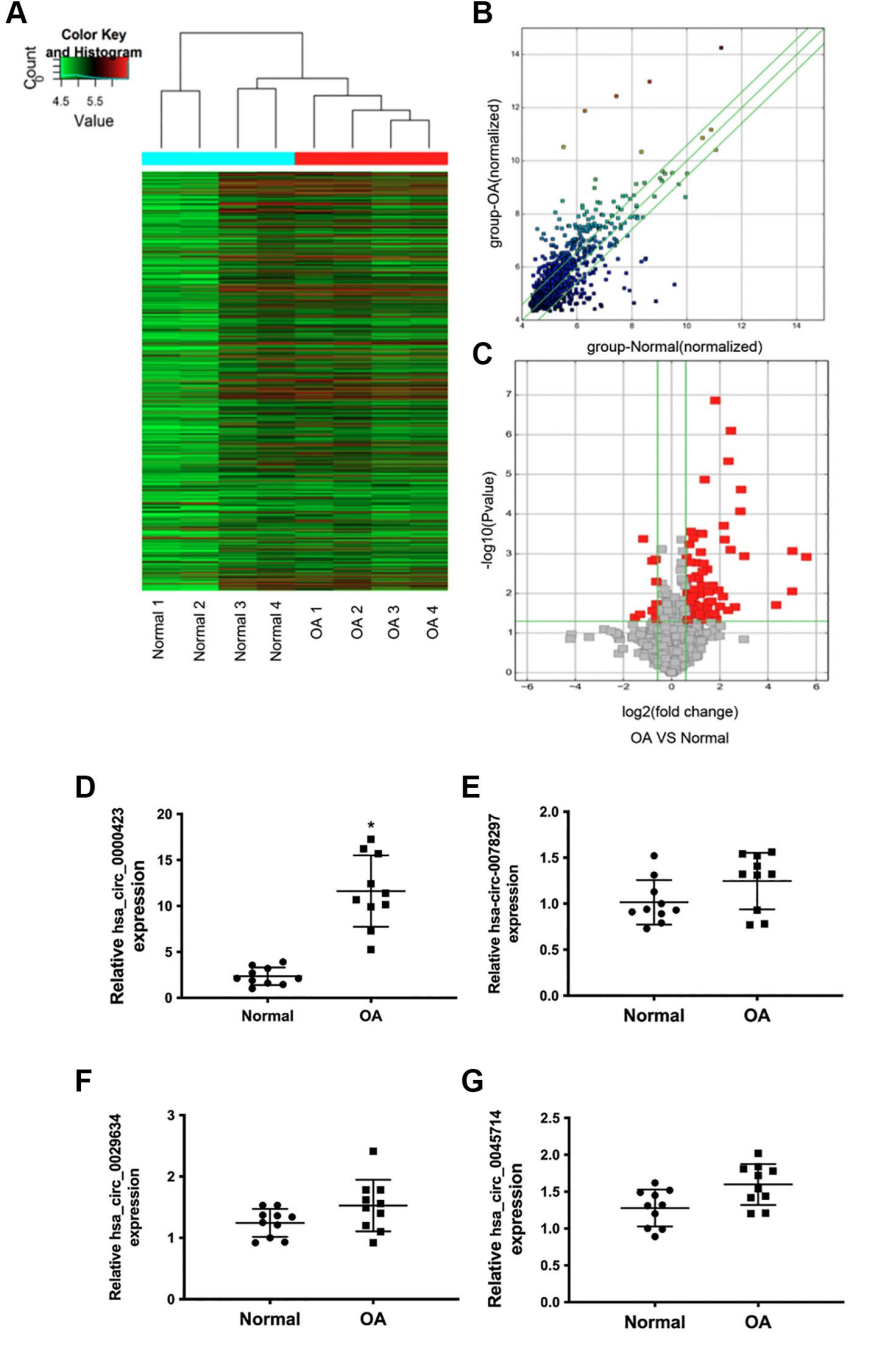
**Differential expression of circRNAs in normal and OA specimens.** (**A**) Hierarchical clustering analysis of the differentially expressed circRNAs between 4 individuals normal and 4 individuals OA specimens; (**B**) Scatter plot of circRNAs expression between normal and OA specimens. The circRNAs below the bottom green line and above the top green line illustrate >2.0-fold changes between the normal and OA specimens. (**C**) Volcano plots of the differentially expressed circRNAs. The red point in the plot represents the differentially down or up-regulated circRNAs with statistical significance. (**D**–**G**) Expression of significant circRNAs in normal and OA specimens.

To evaluate the different circRNAs expression between control and OA tissues, Microarray analysis was performed. As shown in Figure 1A, 1B, Hierarchical clusters and scatter plots displayed the circRNAs expression between control and OA tissues, meanwhile, the significant differences between the two groups were shown in volcano plots filtering (Figure 1C). Based on the Microarray data analysis, 11 circRNAs were down-regulated and 101 circRNAs were up-regulated when compared to control tissues (Supplementary Material). According to the microarray data, we chose some circRNAs which showed significant changes among different circRNAs expressions to further confirm the significant changes among different circRNAs expressions with T-qPCR. The results showed that the expression of circ_0000423 was significantly increased in OA tissues (*p* < 0.05) (Figure 1E–1G).

### Expression of Circ_0000423 is up-regulated in OA tissues and IL-1β induced chondrocyte

**Figure 2:**
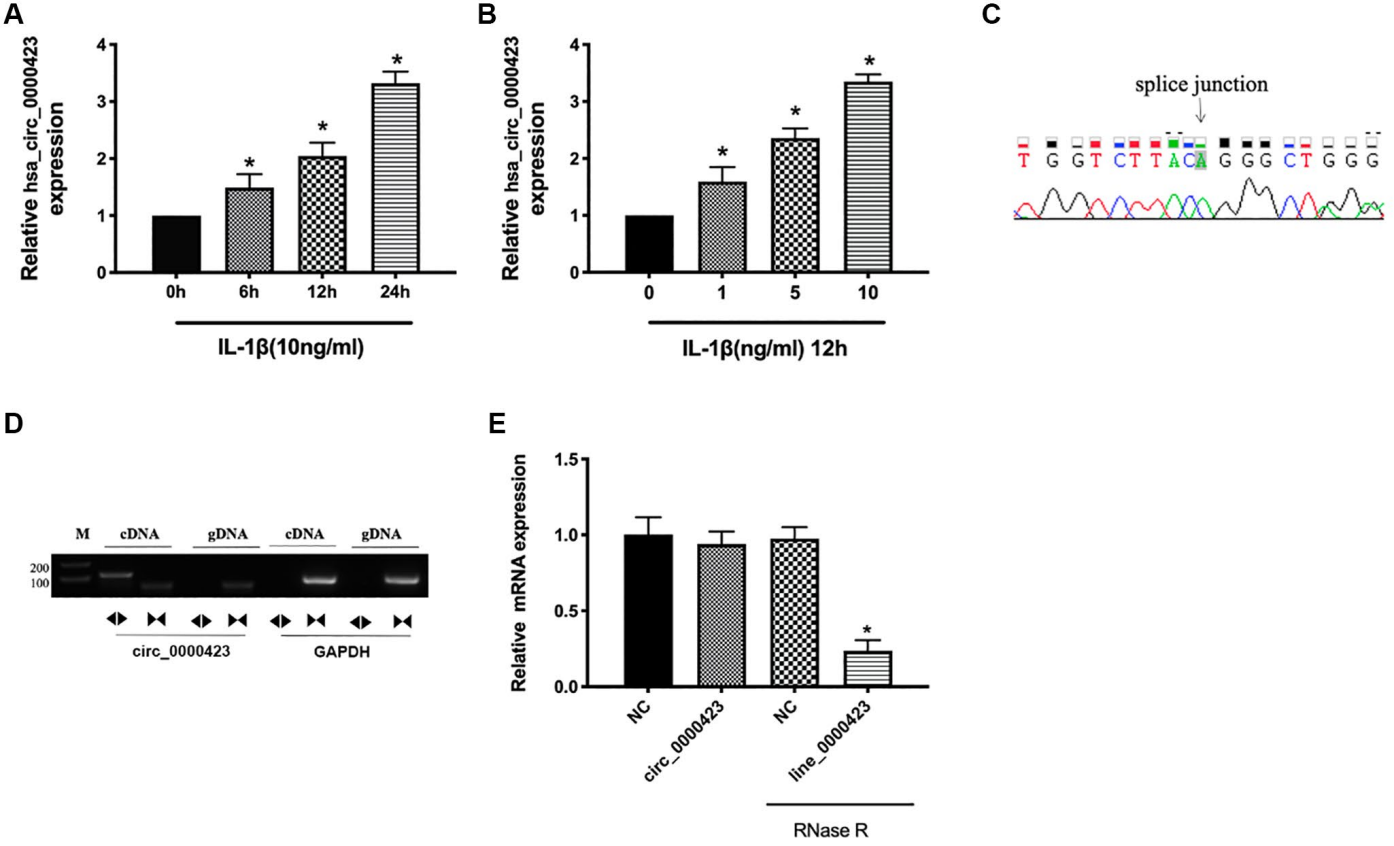
**Expression of Circ_0000423 in cartilage and chondrocyte.** (**A**) Expression of Circ_0000423 in chondrocyte with or without IL-1β induced at different time point; (**B**) Expression of Circ_0000423 in chondrocyte with or without IL-1β induced at different concentrations; (**C**) Sanger sequencing was used to illustrate the presence of Circ_0000423; (**D**) The RT-PCR was used to confirm the presence of Circ_0000423. Divergent primers amplified Circ_0000423 from cDNA, but not from genomic DNA; (**E**) The expression of Circ_0000423 and Line_0000423 mRNA treated with or without RNase R was measured by RT-qPCR; Data are presented as mean ± SEM. ^*^P < 0.05 compared with the control group.

To investigate the Circ_0000423 expression further, RT-qPCR was used to IL-1β induced chondrocytes. The previous results confirmed that Circ_0000423 was significantly increased in OA tissues (*p* < 0.05) (Figure 1D), and similar results was analyzed in IL-1β induced chondrocyte compared with the normal chondrocyte (Figure 2A, 2B). Sanger sequencing of the RT-qPCR products confirmed the back splice junction of Circ_0000423 (Figure 2C); the presence of Circ_0000423 was amplified by divergent primers from cDNA, but not from genomic DNA (Figure 2D). Furthermore, the RNase R was used to confirm the cyclic structure of Circ_0000423 (Figure 2E).

### Circ_0000423 could be directly targeted by miRNA-27b-3p

**Figure 3:**
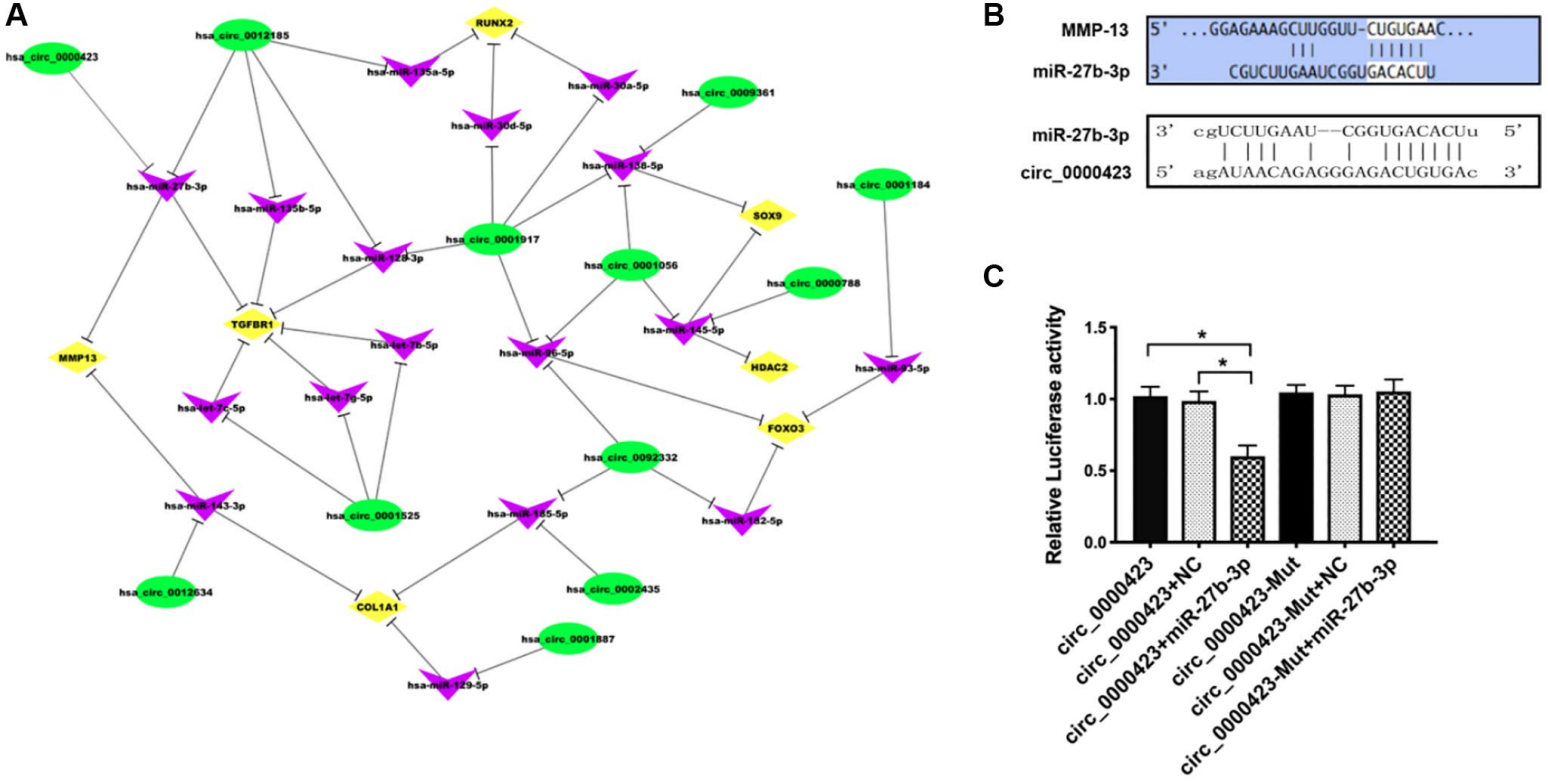
**Circ_0000423 directly targeted by miRNA-27b-3p.** (**A**) circRNAs-miRNA-mRNA network (**B**) Targeted miRNA-27b-3p relationship between Circ_0000423 and MMP-13; (**C**) Luciferase reporter gene assay confirmed the targeted relationship between Circ_00004234 and miRNA-27b-3p. Data are presented as mean ± SEM. ^*^P < 0.05.

According to the microarray data, some circRNAs were chosen to predict the targets. The results of gene co-expression networks showed that Circ_0000423 could be directly targeted by miRNA-27b-3p (Figure 3A). Moreover, the sequences of Circ_0000423 3′-UTR could match the miRNA-27b-3p target through bioinformatics algorithms analysis (Figure 3B). Meanwhile, Luciferase assay confirmed results which Circ_0000423 bind to miRNA-27b-3p. Circ_0000423-psiCHECK-2 vector and miRNA-27b-3p mimic were co-transfected into HEK-293 cells, the results of luciferase activity were significantly lower compared with the control group, however, after miRNA-27b-3p binding sites on Circ_0000423 were mutated, the results could be reversed (Figure 3C). These results demonstrate that Circ_0000423 could be directly targeted by miRNA-27b-3p and act as a miRNA-27b-3p sponge.

### Functions of Circ_0000423 in OA by targeting miRNA-27b-3p expression as a ceRNA

**Figure 4:**
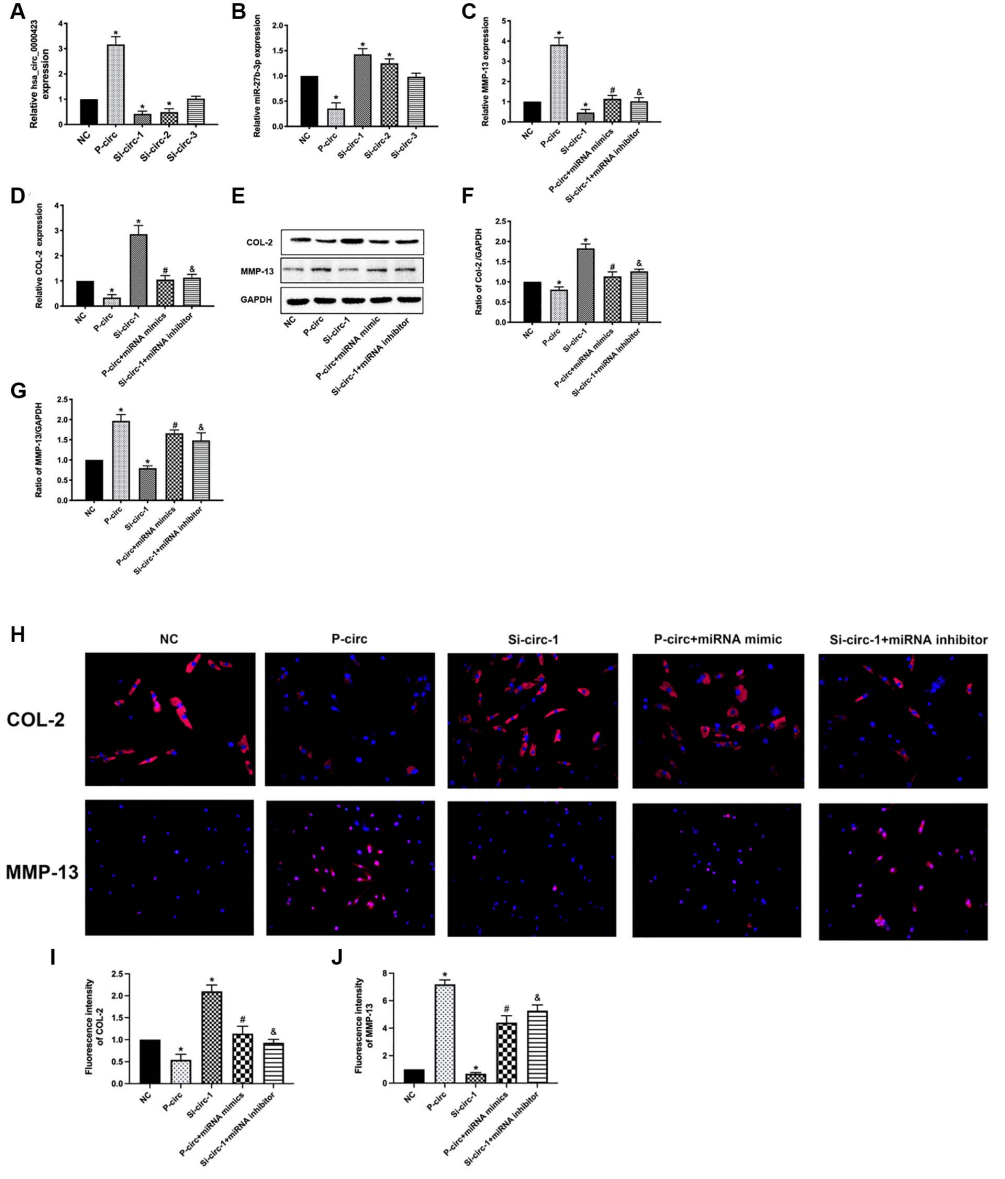
**Functions of Circ_0000423 in OA by targeting miRNA-27b-3p expression as a ceRNA.** (**A**) The mRNA expression of Circ_0000423; (**B**) The mRNA expression of miRNA-27b-3p; (**C**) The mRNA expression of MMP-13; (**D**) The mRNA expression of COL-2; (**E**) The proteins expression of MMP-13 and COL-2; (**F**, **G**) The relative protein expression of COL-2 and MMP-13are demonstrated with bar graphs. (**H**) The relative protein expression of COL-2 and MMP-13 are demonstrated with Immunofluorescence analysis. (**I**, **J**) The relative fluorescence expression of COL-2 and MMP-13 are demonstrated with bar graphs. P-circ: overexpression of Circ_0000423; si-circ-1-3: siRNA against Circ_0000423; Data are presented as mean ± SEM. ^*^P < 0.05 compared with the NC group, ^#^*P* < 0.05 compared with the p-circ group, ^&^*P* < 0.05 compared with the si-circ-1 group.

To analyze whether the functions of Circ_0000423 in OA were through targeting miRNA-27b-3p, we measured the mRNA and protein expression of MMP-13 and collagen II (COL-2) in chondrocytes by co-transfecting relative Circ_0000423 and miRNA-27b-3p, respectively. As shown in Figure 4, we overloaded the Circ_0000423 expression (p-circ) into chondrocytes, which decreased the expression of miRNA-27b-3p and collagen II, and this rate could be reversed by knocking down the Circ_0000423 expressions (si-circ1-2). However, the opposite results were found on the expression of MMP-13. In addition, co-transfection with miRNA-27b-3p mimic, the effects of p-circ_ 0000423 on the expression of MMP-13 and collagen II could be rescued. Besides, co-transfection with miRNA-27b-3p inhibitor, the effects of si-circ_0000423 on the expression of MMP-13 and collagen II were also shown to be rescued. These results demonstrate that Circ_0000423 could affect the expression of MMP-13 and collagen II by targeting miRNA-27b-3p expression as a ceRNA in OA.

### Circ_0000423 regulates OA progression in ACLT (anterior cruciate ligament transection) mice

**Figure 5:**
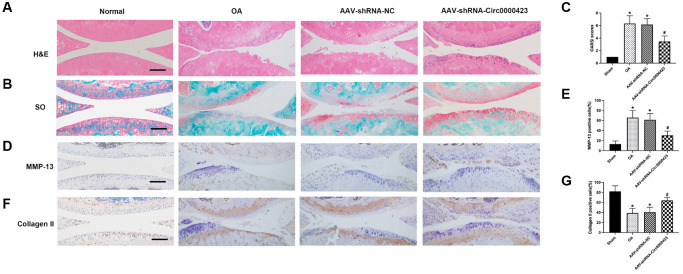
**Circ_0000423 regulates the progression of OA in ACLT mice.** (**A**, **B**) Histological analysis of OA was measured by HE staining and Safranin O staining. (**C**) OARSI scores were used to measure the progression of OA. (**D**–**F**) Immunohistochemical analyses of MMP-13 and Collagen II in sagittal sections of the medial condyle. (**E**–**G**) Quantification of MMP-13 and Collagen II-positive cells. *P* values were computed vs. controls group^*^ or ACLT group^#^; (^*^, ^#^) *P* < 0.05. (Scale bar = 50 μm).

To investigate cartilage surface histopathological changes after intra- Circ_0000423 articular injection in ACLT mice, we used the HE (Figure 5A) and Safranin O-fast green (Figure 5B). The ACLT groups showed more severe degenerative OA changes than the control group, including articular cartilage destruction and erosion, articular surface fibrillation, and osteophyte formation (Figure 5A, 5B). These modifications, however, were inhibited following shRNA-Circ 0000423 treatment. Moreover, the Osteoarthritis Research Society International (OARSI) grade scores results were consistent with histological analyses (Figure 5C). Meanwhile, immunohistochemistry analyses revealed MMP-13 and collagen II expressions in articular cartilage. The percentage of chondrocytes positive for MMP-13 was significantly lower in the shRNA-Circ_0000423 group compared to the ACLT, but it was much higher in the ACLT (Figure 5D–5G). Altogether, these results demonstrated that the shRNA-Circ_0000423 intra-articular injection can slow down OA progress by extracellular matrix (ECM) metabolic homeostasis maintenance and showed its therapeutic potential for OA.

### Silencing of Circ_0000423 suppresses bone resorption induced by ACLT *in vivo*

**Figure 6:**
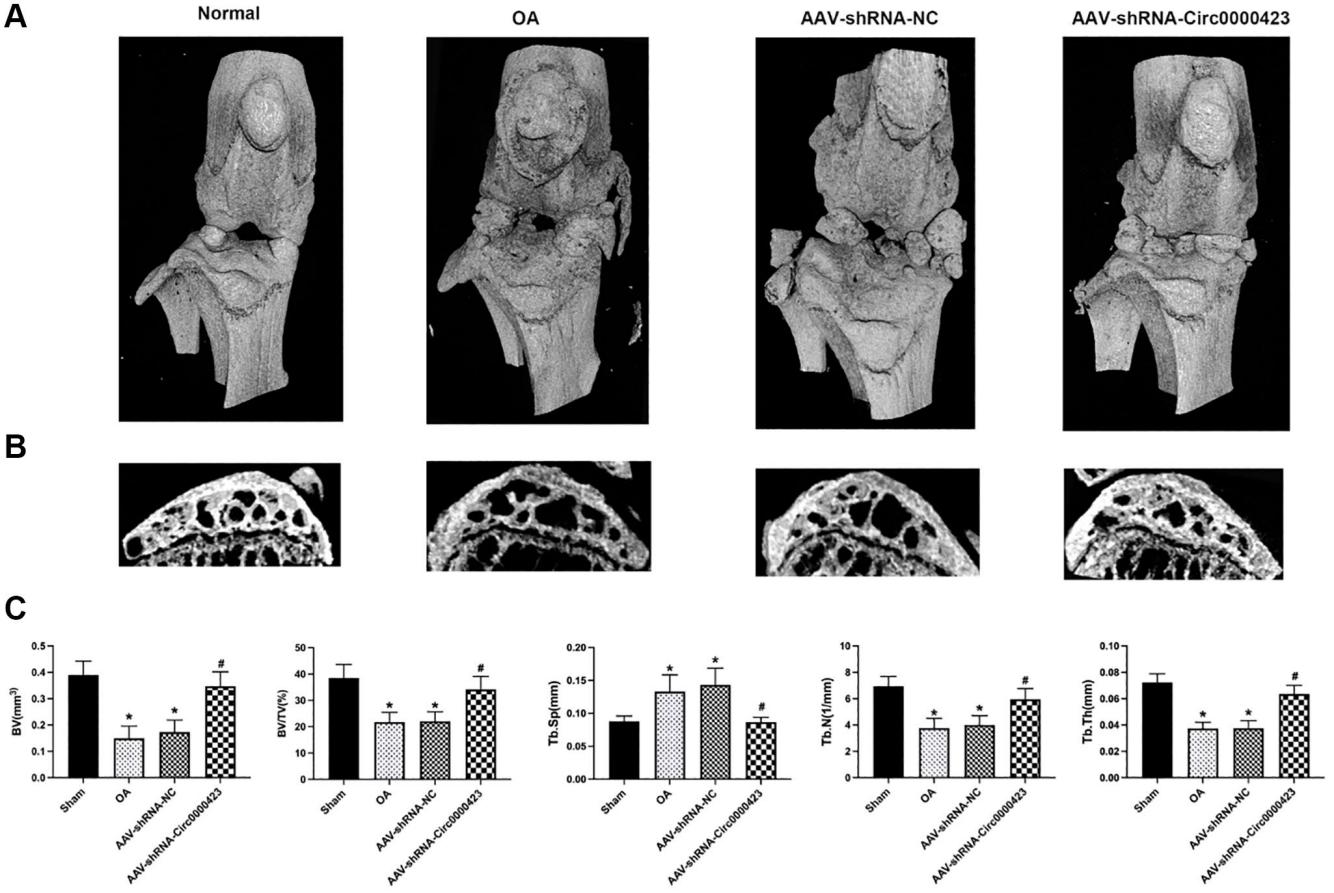
**Silencing of Circ_0000423 suppresses bone resorption induced by ACLT *in vivo*.** (**A**) Three-dimensional micro-CT images of frontal views of the knee joints at 8 weeks after the operation. (**B**) Sagittal views of medial compartment subchondral bone. (**C**) Quantitative analysis of BV, BV/TV, Tb.Sp, Tb.N and Tb.Th. *P*-values were computed vs. controls group^*^ or ACLT group^#^; (^*^, ^#^) *P* < 0.05. (Scale bar = 1 mm).

In the OA initial period, bone loss is crucial for bone remodeling [14]. To illustrate Circ_0000423 effects on OA subchondral bone remodeling, it was intra-articular injected shRNA-Circ_0000423 in mice after ACLT. When compared to the Sham group, bone resorption caused by increased osteolysis in the tibia subchondral bone was significantly higher in the ACLT-induced OA group (Figure 6A, 6B). However, after shRNA-Circ_0000423 treatment, bone resorption decreased and led to increasing bone mass in the subchondral bone and joint bone integrity. This indicated that shRNA-Circ_0000423 treatment can reduce osteolysis *in vivo*. Meanwhile, the tibial plateau subchondral bone volume (BV) in the ACLT (0.15 ± 0.46 mm3) was lower compared to the Sham group (0.39 ± 0.05 mm3; *p* < 0.05). However, after shRNA-Circ_0000423 treatment, BV significantly increased (0.34 ± 0.55 mm3, *p* < 0.05). Moreover, other bone-related parameters (Tb. N, BV/TV, Tb. Sp and Tb. Th) further confirmed the shRNA-Circ_0000423 bone destruction prevention effect *in vivo* (Figure 6C).

## DISCUSSION

OA is one of the most frequent diseases to results in joint pain and affects joint functions [15, 16]. To date, there has been no effective approach to recommend OA treatment in clinical situations. Hence, novel in-depth research in the pathogenesis mechanisms of OA is a vital way to find new effective therapeutics for the treatment of OA. Recently, some research had been shown that circRNAs were the new regulatory molecules in OA [8, 9, 17]. Therefore, in our study, the microarray and bioinformatics technology was used to analyze different circRNAs expressions between the OA and normal cartilage, which found that 11 circRNAs were down-regulated and 101 circRNAs were up-regulated compared with normal cartilage. Among these different circRNAs expressions, Circ_0000423 was chosen, which showed significant changes between two groups, for further research. And our further results showed that Circ_0000423 could be directly targeted by miRNA-27b-3p as a ceRNA to regulate the expression of MMP-13 and collagen II in OA.

CircRNAs, which are made up of introns or exons and formed through gene rearrangement or non-linear mode of RNA splicing, are widely present in the human cell [18, 19]. They are more stable and more conserved compared with the other noncoding RNAs, which lead to potentially be used as new diagnostic molecular biological markers and therapeutic targets [20, 21]. Recently, many studies had shown that circRNAs exert the regulation functions in multiple diseases, such as cardiovascular disease [22], lung cancer [23], Alzheimer’s disease [24], and neurological disorders [25]. Based on the microarray and bioinformatics analysis, we identified 11 down-regulated circRNAs and 101 up-regulated circRNAs in OA cartilage, and we then used clinical OA and normal cartilage to verify the expression of Circ 0000423. Moreover, the Circ_0000423 expression was also verified by IL-1β induced human chondrocytes, because IL-1β is an original factor to regulate the inflammatory response in OA progression [26]. These results demonstrated that circRNAs exerting the regulation functions in OA and Circ_0000423 may be the most important one among the above circRNAs.

Previous researches had illustrated that circRNAs may have multiple biological functions, such as regulating gene expression through “sponges” combined with miRNA [6, 22], encoding proteins through the non-canonical process [27, 28], or binding proteins through combining or sequestering other RNA [29]. The most important discovery function for circRNA is that it can exert the “sponges” functions to bind miRNA to affect the miRNA expression and regulate the mRNA expression at the end. Wang et al., found that circ-4099 could act as a “sponge” to bind miR-616-5p and regulate Sox9 against intervertebral disc degeneration [30]. Shen et al., demonstrated that circSERPINE2 also acted as a sponge to combine with miRNA-1271-5p and affected the process of OA eventually [8]. In our present research, we predicted a circRNA_miRNA-mRNA network according to the analysis of the bioinformatics algorithm. From our circRNA_miRNA-mRNA network results, we found that Circ_0000423 may target miRNA-27b-3p. To further confirm the target relationship between Circ_0000423 and miRNA-27b-3p, the luciferase assay was used. The results showed that Circ_0000423 could be directly targeted by miRNA-27b-3p and acted as a miRNA-27b-3p sponge.

Collagen II (COL-2) is the main constituent of the cartilage extracellular matrix (ECM) [31], while MMP-13 is the most critical enzyme to degrade collagen II, which is directly related to OA [32]. The previous report found that miRNA-27b-3p interacts with the 3′UTR of MMP-13 mRNA and plays a key role in the OA progression [13], and the current result showed that Circ_0000423 could be directly targeted by miRNA-27b-3p. However, Circ_0000423 binds to miRNA-27b-3p whether can regulate the process of OA is still unknown. Therefore, we analyzed the mRNA ofMMP-13 and collagen II expressions in chondrocytes after co-transfecting relative Circ_0000423 and miRNA-27b-3p, respectively. The results showed that Circ_0000423 could affect the MMP-13 and collagen II expressions by targeting miRNA-27b-3p expression as a ceRNA in OA.

Intra-articular local injections of drugs such as hyaluronic acid, platelet-rich plasma, and corticosteroids have been widely employed in the treatment of OA [33, 34]. When compared to systemic delivery, intra-articular local injections have many advantages, including a reduction in extra-articular adverse effects [35, 36]. Previous studies have confirmed cirRNAs importance. Shen et al., [8] reported that cartilage surfaces in ACLT-induced OA rabbits improved after the injection of CircSERPINE2. Zhou et al., [37] reported that silencing CircCDH13 alleviated OA by using AAV intra-articular injection *in vivo* OA mice model. In accordance with previous results, we used the ACLT method to generate the OA mouse model before injecting shRNA-Circ 0000423 intra-articularly. Our findings demonstrated that shRNA-Circ 0000423 treatments slowed OA advancement, reduced MMP-13 expression, and raised collagen II expression in articular cartilage, indicating a potential strategy to limit OA progression. Furthermore, increasing evidence has connected subchondral bone thickness to articular cartilage destruction [38]. In the early stages of OA, osteoclast activity increases, creating an imbalance between bone production and resorption and, eventually, subchondral bone loss [39, 40]. Subsequently, subchondral plate densification will happen, which could finally lead to subchondral sclerosis, osteophytes formation, and cartilage loss in the late OA period [14]. Surprisingly, evidence proved that several circRNAs can regulate subchondral bone abnormal remodeling [8, 41, 42]. In this study, results showed that under shRNA-Circ_0000423 treatment, bone resorption decreased, then results in increased subchondral bone mass and joint bone integrity, which indicated that this treatment could reduce osteolysis.

## CONCLUSION

**Figure 7:**
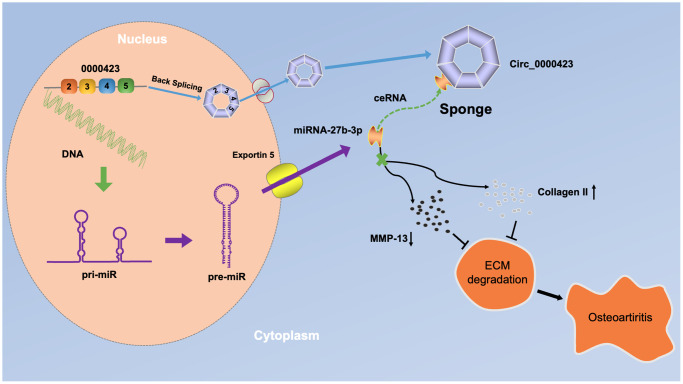
A schematic diagram of the working hypothesis for circ_0000423 in osteoarthritis.

A total of 11 down-regulated circRNAs and 101 up-regulated circRNAs were identified in OA cartilage and confirmed that Circ_0000423- miRNA-27b-3p- MMP-13 axis could affect the pathogenesis of OA (Figure 7). These findings might lead to a novel target for diagnostic molecular biological markers and potential OA treatments.

## MATERIALS AND METHODS

### Clinical specimens

OA cartilage was obtained from knee joints undergoing total knee arthroplasty operation (*n* = 4, age 62–70 years), and normal articular cartilage specimens were isolated from patients with femoral neck fractures (*n* = 4; ages 60–68 years) with no history of OA, who received total hip arthroplasty surgery. All of the patients gave their informed permission. The Ethics Committee on Human Experimentation at Sun Yat-Sen University’s First Affiliated Hospital in China approved the study (IRB: 2011011). Table 1 summarizes the patient information.

**Table 1:** Selected patient characteristics.

**No**	**Gender**	**Age**	**Diagnosis**	**Operation**	**Material position**	**Classification**
1	M	61	Right femoral neck fractures	THA	Right caput femoris	Normal cartilage
2	F	67	Left femoral neck fractures	THA	Left caput femoris	Normal cartilage
3	M	68	Right femoral neck fractures	THA	Right caput femoris	Normal cartilage
4	F	64	Left femoral neck fractures	THA	Left caput femoris	Normal cartilage
5	M	63	Right knee Osteoarthritis	TKA	Right Knee	OA cartilage
6	F	65	Both knee Osteoarthritis	TKA	Right Knee	OA cartilage
7	M	66	Both knee Osteoarthritis	TKA	Left Knee	OA cartilage
8	F	68	Left knee Osteoarthritis	TKA	Left Knee	OA cartilage

### Microarray and bioinformatics analysis

Specimens were analyzed by the Arraystar Human circRNA Array V2 analysis, and then total RNA from each sample was analyzed by the Nanodrop ND-1000. The sample preparation and microarray hybridization were performed according to Arraystar’s standard protocols [43, 44]. In brief, total RNAs were digested with RNase R (Epicentre, Inc.) to remove linear RNAs and enrich circular RNAs. Then, the enriched circular RNAs were measured by Arraystar Human circRNA Array V2 (8x15K, Arraystar). The Agilent Scanner G2505C was used to scan the array images, which were then extracted using Agilent Feature Extraction software (version 11.0.1.1). The R software limma package was used to do quantile normalization and further data processing. The circRNA data, as well as common target mRNAs and miRNAs of the circRNAs, were used to create the mRNA-miRNA-circRNA network. The circRNA/miRNA/mRNA interaction was predicted with Arraystar’s homemade miRNA target prediction software according to miRanda [11] and TargetScan [45].

### Primary chondrocyte isolation and cell culture

Primary chondrocyte isolation was isolated from articular cartilage tissues [12]. In brief, the articular cartilage tissues were sliced into small pieces less than 1 mm^3^ and then stirred to sequential digestion in pronase (90 min) and collagenase P (12 h) at 37°C. Primary articular chondrocytes were maintained in DMEM/F12 (Gibco Life Technology, USA) contained with 5% FBS, 100U/ml penicillin, and 100U/ml streptomycin at 37°C in 5% CO_2_ humidified atmosphere. Every two days, the medium was changed. The following investigations used first-passage chondrocytes. The chondrocytes were treated with IL-1β (PeproTech, China) according to the experiment design.

### Plasmid construction

The construction of circRNA over-expression and Luciferase-hsa_circ_0000423 plasmids were synthesized by Obio Technology (Shanghai, China). The construct method was carious out according to previously described [46]. The resulting construct (pcDNA3.1(+)-S-hsa_circ_0000423) was verified by direct sequencing with the hsa_circ_0000423-F (CGCAAATGGGCGGTAGGCGTG) and hsa_circ_ 0000423-R (TAGAAGGCACAGTCGAGG) primers. The resulting construct (pMIR-REPORT Luciferase-hsa_circ_0000423(WT)) was verified by direct sequencing with the hsa_circ_0000423-F(GCTCAT AGGCCGGCATAGACGCGTCCCTGTAAGACCAGTAATAAATTTCT) and hsa_circ_0000423-R (AAA TAAAAGATCCTTTATTAAGCTTCTGGGTGGCCTTTTCCAACT) primers. The resulting construct (pMIR-REPORT Luciferase-hsa_circ_0000423(MUT)) was verified by direct sequencing with the hsa_circ_ 0000423-F(AAAAATTAATCAGTTTTGAGTGGCA GCGGCGATGCTACTCTTTTGAACTGTCAAGTT) and hsa_circ_0000423-R(GAGTAGCATCGCCGCTG CCACTCAAAACTGATTAATTTTTGTGATATATAATAAGTGG) primers.

### RNA transfection

Chondrocytes transfection was used by Lipofectamine 2000 (Gibco Life Technology, USA) based on the manufacturer’s instructions [47]. PcDNA3.1-Circ_0000423, si-Circ_0000423-1, and si-Circ_0000423-2 (small interfering RNAs for Circ_0000423) were synthesized by Obio Technology (Shanghai, China); miR-27b-3p mimics, miR-27b-3p inhibitor, and negative control (NC) were purchased from RiboBio Technology (Shanghai, China).

### Quantitative real-time polymerase chain reaction (qRT-PCR)

Trizol reagent was used to extract total RNA from chondrocytes and cartilage tissues (Invitrogen, USA). To digest RNaseR, the samples were heated to 37°C for 25 minutes and digested with RNaseR (2 U/mg, Epicenter). For circRNA and mRNA analysis, the total RNA was reversed via the First cDNA Synthesis Kit (Takara, Japan), and RT-PCR was carried outperformed using the SYBR green system (Toyobo, Japan). For miRNA analysis, total RNA was reversed using the miRNeasy Mini Kit (QIAGEN, CA, USA), and RT-PCR was performed using the YBR Premix Ex TaqTM II (Takara, Japan). The circRNA and miRNA expressions were normalized against U6 expression and the mRNA expression was normalized against GAPDH expression. Data were quantified by the 2^−ΔΔCT^ methods. Primer sequences are displayed in Table 2.

**Table 2:** Primers used for target amplification in this study.

**Name**	**primer**	**Accession number**	**Sequence (5′–3′)**
COL-2	forward	NM_033150.3	GCACCTGCAGAGACCTGAAAC
	reverse		GCAAGTCTCGCCAGTCTCCA
MMP-13	forward	NC_000075.6	TCCTGATGTGGGTGAATACAATG
	reverse		GCCATCGTGAAGTCTGGTAAAAT
GAPDH	forward	NC_000012.12	GCACCGTCAAGGCTGAGAAC
	reverse		TGGTGAAGACGCCAGTGGA
miR-27b-3p	forward	MIMAT0000419	CGTCTTGAATCGGTGACACTT
circ_0000423	forward		GCAGCAGGCTAGAAAAGGATG
	reverse		GCCATTGGCTCTGCATTTCA
U6	forward		GCTTCGGCAGCACATATACTAAAAT
	reverse		CGCTTCACGAATTTGCGTGTCAT

### Western blot analysis

Western blot experiments were carious out according to previously described [48]. To conclude, total protein was extracted with lysis buffer (Beyotime, China) and measured with a BCA Protein Assay Kit (Beyotime, China). Then, 50 μg of protein was separated on 10% SDS-PAGE and transferred onto 0.2 μm polyvinylidene difluoride membranes (Millipore, USA). The membranes were incubated with primary antibodies against MMP-13 (1:1000 dilutions, Abcam, UK) and collagen II (1:1000 dilution, Abcam, UK) overnight at 4°C after blocking with 5% non-fat milk for 30 min. GAPDH was used as a control. Then, the membranes were incubated with respective secondary antibodies conjugated with HRP (1:1000, cell signaling technology) for 60 min at room temperature. Band intensity was captured using chemiluminescence imaging reagents (Bio-Rad, USA) were used to measure Band intensity, and ImageJ software (National Institutes of Health, Bethesda, MD) was used to analyze the data.

### Immunofluorescence

Immunofluorescence experiments were carious out according to previously described [49]. Chondrocytes from various treatment groups were washed twice in PBS before being used to make cell smears. The cells were fixed for 30 minutes at room temperature with 4 percent paraformaldehyde, washed twice with PBS, and then treated for 15 minutes at room temperature with 0.1% Triton-X 100 to render them permeable. After washing the permeate, Collagen II (1:200 dilution, Abcam, UK) and MMP-13 antibody (1:200 dilution, Abcam, UK) were added and the reaction was incubated overnight at 4°C. After washing out the first antibody, a fluorescently labeled secondary antibody (1:200, Abcam, UK) was added and incubated at room temperature for 1 hour before being washed three times with PBS. After adding DAPI staining and washing out the staining solution the cells were observed under the fluorescence microscope (Olympus DP80, Japan).

### Dual-luciferase assays

The linear sequence of Circ_0000423 was cloned into the psiCHECK-2 vector (Promega, China), and the potential binding sites of miR-27b-3p were mutated. pcDNA3.1 empty vector (EV) with either Circ_0000423 WT or Circ_0000423 MUT was co-transfected into HEK-293 cells with miR-27b-3p mimics or negative control (NC) based on the manufacturer’s instructions of Lipofectamine 2000 (Gibco Life Technology, USA). The cell lysates were collected 48 hours after transfection. Renilla luciferase activity was measured using the Dual Glo Luciferase Assay System (Promega).

### OA mouse model establishment by anterior cruciate ligament transection (ACLT)

Ten weeks-old male C57 BL/6 mice were randomly assigned into four groups. Each group consisted of 8 mice: Sham, ACLT, AAV shRNA-NC and AAV shRNA-Circ_0000423. The AAV shRNA-NC and AAV shRNA-Circ_0000423 were constructed by HANBIO (Shanghai, China). Then, 3% pentobarbital sodium (35 mg/kg) was injected intraperitoneally for general anesthesia. The process was carried out in an aseptic condition. The mice were acclimated for one week before the operation. We used ACLT to create the mouse OA model, as previously described [50]. The AAV shRNA-N Cand AAV shRNA-Circ_0000423 groups were intra-articularly injected with 10 μL of AAV shRNA-NC and AAV shRNA-Circ_0000423 through the medial parapatellar area at one week after the operation. The Sham and ACLT groups were intra-articularly injected with the same volume of vehicle. After 8 weeks, all mice were put to death. We harvested knee joints and fixated them in 4% paraformaldehyde for follow-up experiments. Animal experiments were carried out with the approval of the Institutional Ethics Committee of Sun-Yat University’s First Affiliated Hospital. All animal studies were carried out following the Institutional Animal Ethics Committee (IAEC) guidelines for the use of laboratory animals.

### Evaluations with micro-CT scanning

Specimens were placed in Eppendorf tubes containing 70% ethanol and scanned using micro-CT (Skyscan 1172, Bruker, Belgium) as previously described [50, 51]. The entire tibial plateaus subchondral bone was set as a region of interest (ROI). We analyzed three-dimensional anatomical structures, including bone volume (BV), bone volume/total tissue volume (BV/TV), trabecular number (Tb. N), thickness (Tb. Th), and separation (Tb. Sp).

### Histological observation and immunohistochemistry

Tissue samples were transferred to 10% EDTA solution for complete decalcification, dehydration, and paraffin embedding. Sagittal planes were cut into a 5-m-thick slice of the medial compartment of the knee joint and stained with hematoxylin and eosin (H&E) and Safranin O-fast green (S&O). The Osteoarthritis Research Society International (OARSI) scoring system was applied as previously described [52].

Immunohistochemistry was used to identify tissue proteins. We sealed the paraffin sections with a serum first and then hatched them with rabbit polyclonal antibody working solution against collagen II or anti-MMP-13(gb12021, gb11247; all 1:100 dilution; Servicebio, PR China) antibodies. They were then incubated with the secondary antibody working solution, which resulted in the formation of the chromogenic reagent color. The number of positively stained cells per specimen was calculated, and three continuous specimens groups were taken from each mice group.

### Statistical analysis

At least three biological replicates were used in each experiment. All data were displayed as mean ± standard deviation (SD). Student’s *t*-test or One-way ANOVA was applied to identify differences between groups. *P* < 0.05 was considered statistically significant. Data analysis was performed with SPSS Statistics 18.0 software (IBM Corp, NY, USA).

### Ethics approval and consent to participate

The research plan approval was obtained from The Ethics Committee on Human Experimentation at The First Affiliated Hospital of Sun Yat-Sen University, China (IRB: [2020]113-1), and complied with The Declaration of Helsinki (2000). All participants signed the informed consent. Animal experiments were performed with the approval of the Institutional Ethics Committee of The First Affiliated Hospital of Sun-Yat University.

### Availability of data and materials

The data used to support the findings of this study are available from the corresponding author upon request.

## Supplementary Materials

Supplementary Material
